# Variability in vascular smooth muscle cell stretch-induced responses in 2D culture

**DOI:** 10.1186/s13221-015-0032-0

**Published:** 2015-08-21

**Authors:** Laura-Eve Mantella, Adrian Quan, Subodh Verma

**Affiliations:** Division of Cardiac Surgery, Keenan Research Centre for Biomedical Science, St. Michael’s Hospital, 8th Floor, Bond Wing, 30 Bond Street, Toronto, ON M5B 1W8 Canada; Department of Pharmacology & Toxicology, University of Toronto, Toronto, ON Canada; Department of Surgery, University of Toronto, Toronto, ON Canada

## Abstract

The pulsatile nature of blood flow exposes vascular smooth muscle cells (VSMCs) in the vessel wall to mechanical stress, in the form of circumferential and longitudinal stretch. Cyclic stretch evokes VSMC proliferation, apoptosis, phenotypic switching, migration, alignment, and vascular remodeling. Given that these responses have been observed in many cardiovascular diseases, a defined understanding of their underlying mechanisms may provide critical insight into the pathophysiology of cardiovascular derangements. Cyclic stretch-triggered VSMC responses and their effector mechanisms have been studied *in vitro* using tension systems that apply either uniaxial or equibiaxial stretch to cells grown on an elastomer-bottomed culture plate and *ex vivo* by stretching whole vein segments with small weights. This review will focus mainly on VSMC responses to the *in vitro* application of mechanical stress, outlining the inconsistencies in acquired data, and comparing them to *in vivo* or *ex vivo* findings. Major discrepancies in data have been seen in mechanical stress-induced proliferation, apoptosis, and phenotypic switching responses, depending on the stretch conditions. These discrepancies stem from variations in stretch conditions such as degree, axis, duration, and frequency of stretch, wave function, membrane coating, cell type, cell passage number, culture media content, and choice of *in vitro* model. Further knowledge into the variables that cause these incongruities will allow for improvement of the *in vitro* application of cyclic stretch.

## Introduction

Vascular smooth muscle cells (VSMCs), in addition to endothelial cells (ECs) and fibroblasts, are one of the three main cell types that compose the blood vessel wall [1, 2]. VSMCs, found in the tunica media, are terminally undifferentiated cells, in that they alter their phenotype based on the surrounding microenvironment. In healthy adult blood vessels, SMCs generally display a contractile, or differentiated, phenotype, characterized by a slow rate of proliferation and the expression of contractile, or smooth muscle cell, markers. These markers include calponin, smooth muscle (SM) alpha actin, SM myosin heavy chain (MHC), and SM22 [3–5]. The expression of these contractile markers is largely regulated by the dimerization and binding of the transcription factor serum response factor (SRF) to CArG elements in the promoter regions of smooth muscle cell-specific genes [4–10]. The contractile nature of VSMCs allows them to regulate myogenic tone, blood pressure, and blood flow within the blood vessel [4]. Conversely, in the context of vascular injury, VSMCs often switch from a contractile to synthetic, or undifferentiated, phenotype, which is characterized by a decrease in the expression of contractile markers. Moreover, synthetic VSMCs display increased rates of VSMC proliferation, migration and extracellular matrix (ECM) remodeling [3–5].

Blood pressure is largely responsible for mechanical stress on the blood vessel wall. During systole, the vessel, and consequently, the VSMCs, experience both longitudinal and circumferential stretch [1]. Under physiological conditions, the aorta undergoes about 10 % circumferential strain during systole [11]. This number increases to about 20 % in conditions of hypertension [12, 13]. The pulsatile nature of blood flow exposes the VSMCs in blood vessels to cyclic mechanical stretch. The elasticity of blood vessels allows them to counteract the perpendicular and longitudinal forces exerted by increases in blood pressure [1, 12]. To adapt to increases in blood pressure, blood vessels undergo vascular remodeling, which encompasses changes in VSMC and EC migration, proliferation and apoptosis, as well as turnover of ECM proteins, to increase their rigidity [1, 2]. This remodeling contributes to the pathogenesis of many vascular diseases.

This review will focus mainly on VSMC responses under *in vitro* conditions and in response to mechanical stress. It will detail the inconsistencies in the available literature, and compare and contrast these findings to the corresponding *in vivo* or *ex vivo* observations. Major discrepancies in data have been noted in mechanical stress-induced proliferation, apoptosis, and phenotypic switching responses, depending on the stretch conditions. These discrepancies derive from variations in stretch conditions such as degree, axis, duration, and frequency of stretch, wave function, membrane coating, cell type, cell passage number, culture media content, and choice of *in vitro* model. Further knowledge into the variables that cause these incongruities will allow for improvement of the *in vitro* application of cyclic stretch.

## Methods

The effects of cyclic stretch on VSMCs and their associated mechanisms have widely studied *in vitro* using the Flexcell® Tension System, and less commonly the STREX Cell Stretching System (Tables 1, 2, 3, 4, 5 and 6). The Flexcell® system applies vacuum pressure to deform elastomer-bottomed cell culture plates, allowing the user to adjust frequency, duration, and degree of stretch to which the VSMCs are subjected [14]. Studies have also been performed using a 3D collagen lattice, to better represent the three-dimensional structure of the blood vessel [15, 16]. Furthermore, researchers have created an *ex vivo* model by statically stretching murine portal vein segments [17]. This technique involves mounting the vein in a test tube containing DMEM culture medium and stretching the vein using 0.3 g weights.

**Table 1:** Cyclic mechanical stretch affects alignment in VSMCs *in vitro*

Study	Cell type	Freq.	Unit	Stretch	Plate	Axis	Time	Wave	Results
Zhu [62]	AoSMC (human)	1 Hz	STREX	10 %	Collagen I	Uniaxial	0–3 h		Alignment ↑
Liu [63]	AoSMC (rat)	0.5–2 Hz	FX-4000	10 %	Collagen I	Equibiaxial	0–12 h		Alignment ↑
Chen [60]	AoSMC (bovine)	1 Hz	FX-4000	10 %	Collagen I		24 h, 48 h	Sinusoidal	Alignment ↑
Standley [59]	AoSMC (rat)	1 Hz	Flexercell	20 %	Collagen I	Uniaxial	48 h		Alignment ↑
Li [61]	AoSMC (bovine)	1 Hz	FX-4000	10 %	Collagen I		0–24 h	Square	Alignment ↑

**Table 2:** Cyclic mechanical stretch affects proliferation in VSMCs *in vitro*

Study	Cell type	Freq.	Unit	Stretch	Plate	Axis	Time	Wave	Results
Standley [59]	AoSMC (rat)	1 Hz	Flexercell	20 %	Collagen I	Uniaxial	48 h		Proliferation ↑
Li [61]	AoSMC (bovine)	1 Hz	FX-4000	10 %	Collagen I		0–24 h	Square	Proliferation ↑
Chahine [24]	AoSMC (rabbit)	1 Hz	FX-4000	20 %			48 h		Proliferation ↑
Chang [23]	AoSMC (rat)	1 Hz	FX-2000	20 %			0–24 h	Sinusoidal	Proliferation ↑
Liu [27]	AoSMC (rat)	1 Hz	Flexercell	5 %, 15 %	Collagen I		2 h		Proliferation ↑
Liu [25]	AoSMC (mouse)	1 Hz	FX-3000	0–25 %			0–60 min		Proliferation ↑
Mata-Greenwood [22]	PASMC (sheep)	1 Hz	FX-4000	20 %	Collagen I	Biaxial	0–24 h		Proliferation ↑
Song [31]	Venous VSMC (rat)	1 Hz	Custom				0–24 h		Proliferation ↑
Song [32]	AoSMC (human)	1 Hz	FX-5000	10 %, 16 %	Collagen I		0–24 h		Proliferation ↑
Morrow [29]	VSMC (rat)	1 Hz	FX-4000	0–15 %			0–24 h	Heart(P)	Proliferation ↓
Guha [28]	VSMC (rat)	1 Hz	FX-4000	0–10 %	Pronectin		0–24 h	Heart(P)	Proliferation ↓

**Table 3:** Cyclic mechanical stretch affects apoptosis in VSMCs *in vitro*

Study	Cell type	Freq.	Unit	Stretch	Plate	Axis	Time	Wave	Results
Cheng [41]	AoSMC (human)	1 Hz	FX-2000	20 %	Collagen I		0–24 h	Sinusoidal	Apoptosis ↑
Su [44]	Portal vein VSMC (swine)	1 Hz	FX-4000	10 %	Laminin		24 h		Apoptosis ↑
Sotoudeh [43]	VSMC (porcine)	1 Hz	Custom	7 %, 25 %		Equibiaxial	0–48 h	Sinusoidal	Apoptosis ↑
Wernig [42]	VSMC (rat)	1 Hz	FX-4000	7 %, 15 %	Collagen I		0–60 min		Apoptosis ↑
Song [32]	AoSMC (human)	1 Hz	FX-5000	10 %, 16 %	Collagen I		0–24 h		Apoptosis ↑
Morrow [29]	VSMC (rat)	1 Hz	FX-4000	0–15 %			0–24 h	Heart(P)	Apoptosis ↑
Guha [28]	VSMC (rat)	1 Hz	FX-4000	0–10 %	Pronectin		0–24 h	Heart(P)	Apoptosis ↑

**Table 4:** Cyclic mechanical stretch affects differentiation in VSMCs *in vitro*

Study	Cell type	Freq.	Unit	Stretch	Plate	Axis	Time	Wave	Results
Yao [51]	AoSMC (rat)	1.25 Hz	FX-4000	10 %	Gelatin	Equibiaxial	24 h		Differentiation ↑
Turczynska [17]	Mouse portal vein		0.3 g weight				5 min		Differentiation ↑
Hu [46]	AoSMC (human)	1 Hz	FX-5000	16 %	Collagen I		0–30 min		Differentiation ↓
Butcher [45]	AoSMC (rat)	1 Hz	Custom	10 %	Collagen I, Fibronectin, Cel-tak	Equibiaxial	48 h	Sinusoidal	Differentiation ↓
Wan [48]	AoSMC (rat)	1.25 Hz	FX-4000	5 %, 15 %	Collagen I		24 h		Differentiation ↓
Rodriguez [47]	AoSMC (rat)	1 Hz	FX-5000	10 %	Collagen I	Uniaxial	0–24 h	Sinusoidal	Differentiation ↓
Song [31]	Venous VSMC (rat)	1 Hz	Custom				0–24 h		Differentiation ↑

**Table 5:** Cyclic mechanical stretch affects secretion of MMP-2 in VSMCs *in vitro*

Study	Cell type	Freq.	Unit	Stretch	Plate	Axis	Time	Wave	Results
Seo [67]	AoSMC (rat)	1 Hz	FX-4000	0–10 %	Pronectin		0–12 h		MMP-2 ↑
Yamashita [68]	AoSMC (rat)	0.5 Hz	STREX	2 %, 5 %, 20 %	Laminin	Uniaxial	48 h		MMP-2 ↑
Grote [69]	AoSMC (mouse)	0.5 Hz	FX-3000	15 %	Collagen I		0–24 h		MMP-2 ↑

**Table 6:** Cyclic mechanical stretch affects migration in VSMCs *in vitro*

Study	Cell type	Freq.	Unit	Stretch	Plate	Axis	Time	Wave	Results
Rodriguez [47]	AoSMC (rat)	1 Hz	FX-5000	10 %	Collagen I	Uniaxial	0–24 h	Sinusoidal	Migration ↑
Chiu [26]	AoSMC (rat)	1 Hz	FX-2000	10 %, 20 %			0–30 h	Sinusoidal	Migration ↑
Li [57]	AoSMC (mouse)	1 Hz	FX-4000	5 %, 15 %, 20 %	Collagen I		0–24 h		Migration ↑
Scherer [56]	Arterial SMC (human)	0.5 Hz	FX-5000	0–13 %	Collagen I		24 h		Migration ↑

### Proliferation

Since abnormal proliferation of VSMCs plays a significant role in the pathogenesis of vascular diseases such as atherosclerosis and hypertension [18–21], it follows that a clear understanding of the effect of cyclic stretch on VSMC proliferation is critical. Generally, cyclic stretch increases the rate of VSMC proliferation under *in vitro* conditions. Sheep and rat VSMCs that had been exposed to 20 % cyclic stretch demonstrated increased expression of positive regulators of proliferation, such as hypoxia-inducible factor-1α, vascular endothelial growth factor and transforming growth factor (TGF)-β1 [22, 23]. Stretch has also been reported to act synergistically with oxidized low-density lipoprotein and norepinephrine to increase rabbit and mouse VSMC proliferation through activation of the extracellular signal-regulated kinase (ERK) pathway [24, 25] which itself has been implicated in 20 % stretch-induced rat VSMC hypertrophy via increased expression of myocardin [26]. Similarly, rat aortic SMCs (RASMCs) exposed to 15 % stretch showed upregulated ERK and Akt activation, and a subsequent increase in insulin-induced cellular proliferation [27].

The previous data are in contrast with those observed by Guha *et al*. [28], who noted that 10 % stretch decreased rat VSMC proliferation and survival via inhibition of glycogen synthase kinase-3β activity. The same group also found in rat VSMCs that 15 % stretch inhibited proliferation through decreased Notch3 receptor expression [29]. However, it is important to note that unique to these studies was the waveform used to stretch the cells. These studies used the Flexcell® Heart(P) waveform, which mimics physiological pressure generated by a beating heart, rather than the more commonly used sinusoidal waveform. Taken together, these results suggest that pressure waveform may have an important impact on VSMC proliferation.

Stretch-induced cellular proliferation is also dependent on the effects exerted by the small non-coding molecules known as microRNAs which silence RNA by base pairing with complementary sequences within mRNA molecules [30]. It was previously found that in rat venous VSMCs, stretch downregulated miR-223 and miR-153, stimulating proliferation via activation of insulin-like growth factor-1 receptor (IGF-1R) [31]. Similarly, 16 % elongation of human aortic SMCs (HASMCs) resulted in enhanced miR-21 expression, which occurred via activation of the transcription factor, activator protein-1, and ultimately increased the rate of cellular proliferation [32]. Collectively, these findings suggest that stretch-induced increases in proliferation may be, at least partially, regulated by non-coding RNAs which poses the notion that other non-coding RNAs, such as long non-coding RNAs, may also play a role in regulating VSMC response to mechanical stress.

The aforementioned studies clearly demonstrate that VSMC responses *in vitro* are highly dependent on the characteristics of the stretch applied and underscore the importance of concomitantly studying VSMC responses to cyclic mechanical stretch under *in vivo* conditions. The spontaneously hypertensive rat (SHR) model is a widely used model mechanical stretch. Data derived from VSMCs isolated from SHR have revealed conflicting proliferating effects in response to mechanical stretch. Aortic VSMCs from SHRs exhibited increased rates of proliferation after being subjected to mechanical stretch [33–36] and this phenomenon was associated with decreased nitric oxide (NO) sensitivity [36], faster cell cycle progression due to increases in cyclin D, E and A expression [35], and increases in mitogen-activated protein kinase (MAPK) activity [33, 34]. In contrast, Arribas and colleagues recorded in carotid artery VSMCs from neonatal SHRs, decreases in PCNA- and BrdU- positive nuclei, both hallmarks of decreased cellular proliferation [37]. The differential results reported with VSMCs from the same rat strain suggest that age and VSMC origins likely pre-determine the stretch-induced responses we measure.

### Apoptosis

Dysregulated apoptosis is also a key contributor to the pathogenesis of various cardiovascular diseases. Increased apoptosis has been linked to atherosclerosis, heart failure, and diabetes [38–40] and mechanical stress has been shown to upregulate VSMC apoptosis [28, 29, 32, 41–43]. Stretch exposure promoted apoptosis through the induction of p53 upregulated modulator of apoptosis in human VSMCs and β_1_-integrin activation of p38 MAPK in rat VSMCs [41, 42]. In porcine VSMCs, increased apoptosis occurred in parallel with activation of the p38 MAPK and c-Jun N-terminal kinase pathways following 25 % stretch [43]. Interestingly there was no significant increase in apoptosis seen with 7 % elongation suggesting that the stretch-dependent apoptotic response may also be dependent on the degree of stretch. In HASMCs, 16 % stretch has been associated with parallel elevations in apoptosis and miR-21 expression [32]. Studies using the Heart(P) pacemaker waveform were consistent with the general finding that stretch increased rat VSMC apoptosis [28, 29].

It is noteworthy that data has also suggested that stretch-induced apoptosis differs in VSMCs depending on their phenotype. 10 % stretch was found to induce apoptosis in differentiated, but not undifferentiated porcine VSMCs [44]. However, it was previously demonstrated that stretch generally induces phenotypic switching from a differentiated to undifferentiated state [45–48], begging the question of whether the differentiated cells responded to the stretching stimulus. The VSMCs used in this study were also derived from veins, instead of the more commonly studied arterial SMCs, which may also have affected apoptotic response.

Characterization of the whole-heart and aortic smooth muscle cell apoptosis in SHR showed increases in apoptosis *in situ* and *in vitro* detection of DNA fragmentation, respectively [49]. Likewise, SMCs from the left ventricle of spontaneously hypertensive rats showed increased apoptosis compared to WKY-derived controls [50]. In contrast, SHR-derived carotid artery SMCs displayed decreased apoptosis, accounted for by increased levels of the anti-apoptotic protein, survivin [37]. Again, the origin of the VSMCs may play a role in the discrepancies in apoptosis data from spontaneously hypertensive rats. Since the aorta has greater elasticity than the carotid artery, this may account for differential responses to mechanical stretch applied to the vascular wall by the pulsatile nature of blood flow and the increased pressure from hypertension.

It is of particular interest that both proliferation and apoptosis seem to be generally upregulated as a result of stretch in VSMC cultures. This may suggest that there is a compensatory mechanism in place to counteract the increase or decrease in cell survival. Future studies aimed at elucidating the mechanisms by which pro- and anti-survival factors interact during conditions of mechanical stress are warranted.

### Phenotype

VSMCs are plastic cells, altering their phenotype based on the surrounding microenvironment [3, 5]. Studies have shown that VSMCs undergo phenotypic switching in response to cyclic mechanical stress [17, 26, 45–48, 51]. RASMCs that had been subjected to 10 % elongation underwent phenotypic switching from the contractile to the synthetic phenotype, characterized by a decrease in alpha smooth muscle actin and calponin [45]. It was also observed that 16 % stretch inhibited miR-145, a positive regulator of the HASMC differentiation stimulator, myocardin, via activation of the ERK pathway [46]. Wan *et al*. [48] discovered that endoplasmic reticulum stress caused by mechanical stress on RASMCs induced alternative splicing and activity of big potassium channels which are involved in regulating vascular tone [52]. Furthermore, the splicing outcome effected adoption of a synthetic phenotype [48]. Of interest, detection of SMC contractile markers in early passage SHR-derived VSMCs demonstrated that these cells display a synthetic phenotype [53].

The above mentioned *in vitro* studies demonstrate the effects of equibiaxial stretch on the modulation of VSMC phenotype. The effects of uniaxial stretch have been assessed *in vitro* by means of either a Flexcell® unit or *ex vivo* by stretching an entire vein segment using a 0.3 g weight [17, 47]. Rodriguez *et al.* [47] demonstrated, using a Flexcell® Tension System, that 10 % uniaxial stretch upregulated NADPH oxidase 1 (Nox1) via myocyte enhancer family 2B (MEF2B). This increased production of reactive oxidative species (ROS), and led to the adoption of a synthetic phenotype in rat aortic SMCs. In contrast to the previously described findings, Turczynska *et al*. [17] found that stretch caused VSMCs to adopt a contractile, or differentiated, phenotype and this was associated with a decrease in the miR144/154 cluster. Notably, however, these researchers used an *ex vivo* model of venous elongation whereby a segment of murine portal vein was stretched with a 0.3 g weight. The *ex vivo* system used in these studies may exemplify the effects of VSMC-EC crosstalk, which will be discussed in greater detail later in this review. Briefly, since VSMCs and ECs are located in close proximity in the intact blood vessel, many studies have aimed to identify the effects of VSMC-EC interactions. For example, VSMCs co-cultured with ECs display differential gene expression, compared to a mono-cultured VSMCs [54]. Thus, the results obtained by Turczynska *et al*. [17] may account for VSMC-EC communication.

The discrepancies in the phenotypic switching data available to date may not be easily resolved solely by stratifying results based on axis of stretch. Yao *et al*. [51] found that 10 % equibiaxial stretch had a positive influence on RASMC differentiation via activation of the TGF-β1 pathway and its consequential upregulation of sirtuin-6 (SIRT6). The contradicting results in the previous study underscore the uncertainty associated with *in vitro* and *ex vivo* models of VSMC stretch. The numerous independent and cooperative pathways outline the need to further characterize the mechanisms through which VSMC phenotype is modulated in conditions of cyclic mechanical stress.

### Migration

As VSMCs adopt their synthetic phenotype, the rates of proliferation and migration increase [3–5]. During atherogenesis and tissue repair after vascular injury, VSMCs migrate from the tunica media to the tunica intima [55]. Since VSMC migration is correlated with the pathogenesis of vascular diseases, studies have been conducted to determine the effects of mechanical stress on migration *in vitro*. Exposure to 24-h 13 % elongation has been shown to activate cellular migration through stimulation of nuclear factor of activated T-cells 5 nuclear translocation in human VSMCs [56]. Similarly, 20 % stretch enhanced rat VSMC migration via ERK-dependent increases in myocardin expression [26]. Furthermore, the protein kinase C δ (PKCδ) pathway was implicated in stretch-dependent migratory response, as bovine aortic SMCs exposed to 15 % stretch experienced enhanced migration [57]. Supporting evidence was obtained using VSMCs from explanted SHR and Wistar Kyoto (WKY) rat aortas. The group discovered that migration in SHR-derived VSMCs was increased compared to WKY rat aorta-derived VSMC controls [33]. Taken together, these data contend that stretch, through many different mechanisms, has a positive regulatory role on VSMC migration.

### Alignment

VSMCs in the vessel wall are arranged in the form of a fibrous helix. In response to mechanical stimuli during development, angiogenesis, and vascular remodeling, VSMCs undergo changes in alignment [58]. The application of 20 and 10 % uniaxial cyclic stretch induced aortic SMC alignment perpendicular to the axis of stretch via activation of the p38 MAPK pathway and the induction of NO synthesis, respectively [59, 60]. Equibiaxial cyclic stretch has also been shown to induce VSMC alignment *in vitro*. This response is attributed to activation of the mTOR/S6 kinase and p38 MAPK pathways, and the increase in Notch3 expression stimulated by stretch-induced ROS production [61, 62].

Interestingly, Liu *et al.* [63] found that stretch-induced alignment was dependent on the frequency of stretch, as 1.25 Hz induced the greatest alignment perpendicular to the axis of stretch, compared to conditions of 0.5, 1 and 2 Hz. Collectively, these studies demonstrate the numerous factors that must be taken into consideration when performing *in vitro* stretch analyses. The frequency, axis and degree of stretch have been shown to play a large role in the VSMC alignment response.

To study VSMC alignment *in vivo*, laser-scanning confocal microscopy was used to determine VSMC orientation in the basilar arteries of SHRs [64]. In contrast to *in vitro* data, VSMCs from WKY rats were uniformly oriented perpendicular to the longitudinal axis of the vessel, whereas those from SHRs exhibited disarray. This major discrepancy outlines the differences between *in vitro* and *in vivo* models of mechanical stretch, and emphasizes the need for more accurate *in vitro* models.

### Vascular remodeling

Vascular remodeling generally occurs as a response to hemodynamic changes in the blood vessel and is often a critical pathophysiological component of many cardiovascular diseases. It involves changes in proliferation, apoptosis, migration, and reorganization of the ECM [65]. Matrix metalloproteinases (MMPs) have been implicated in the decomposition of the ECM and are largely involved in vascular remodeling [66]. Researchers have established that, under conditions of mechanical stress, cultured VSMCs from rat and mouse experienced increases in the expression and activity of MMP-2, which was modulated by the Akt pathway, periostin/focal adhesion kinase (FAK) system and ROS production [67–69].

Increased expression and activity of MMPs in response to stretch have been observed in VSMCs from the aortas of SHR. These cells were found to have higher MMP-9 levels and activity relative to those from aortas of normotensive controls [33]. Similarly, left ventricular tissue from these rats had higher levels of both MMP-2 and MMP-9 activity [70]. Human saphenous vein grafts subjected to uniaxial stretch to a maximum elongation of 150 % of their resting length for 5 s demonstrated enhanced pro-MMP-9 expression and MMP-2 activity 3–5 days after later [71]. In contrast, Lin *et al*. who studied Dahl salt-sensitive rats did not find any changes in MMP-2 expression [72].

### Potential biomarkers

In order to more accurately assess *in vitro* VSMC stretch response; stretch-related variables such as degree, duration, axis and frequency of stress, must remain consistent. Furthermore, passage number, calibration of instruments, membrane coating, and wave function used, must be carefully documented. Equally important however is to define and standardize universal biomarkers for stretch in VSMCs.

#### ROS

One molecular event that seems to be involved in a number of VSMC stretch responses is the increased production of ROS [22, 47, 62, 67, 69, 73–75]. Human and rat aortic SMCs subjected to 10 % elongation for 24 h exhibited enhanced ROS generation [47, 62, 73] implicating ROS in both VSMC stretch-responsive alignment [62, 73] and adoption of the synthetic phenotype [47]. Studies by Montenegro *et al*. and Dick *et al.* suggested a Nox-4-dependent increase in ROS in response to stretch in human aortic and rat pulmonary artery SMCs (PASMCs), respectively [73, 75]. Another member of the Nox family, Nox-1, has also been implicated in stretch-dependent increases in ROS production [47, 69]. Specifically, Rodriguez *et al*. proposed Nox-1-mediated phenotypic switching to a synthetic phenotype in RASMCs subjected to 10 % uniaxial elongation for 24 h [47]. It was also found that mechanical stretch increased Nox-1 production, which lead to overall increases in MMP-2 expression in mouse aortic SMCs subjected to 15 % elongation, suggesting a role for ROS in stretch-induced vascular remodeling [69]. Similar findings were proposed by Seo *et al*. in RASMCs subjected to 10 % elongation [67]. Taken together, these findings suggest that increased ROS production may be a characteristic of stretched VSMCs, and this increase may be Nox-dependent. If so, Nox-1 and Nox-4 may act as potential biomarkers of mechanical stretch in VSMCs.

#### TGF-β1

TGF-β1 is a positive regulator of proliferation that is upregulated in sheep PASMCs that have been exposed to 20 % cyclic stretch. Yao *et al*. [51] discovered a role for TGF-β1 in promoting RASMC differentiation via activation of sirtuin-6 (SIRT6) in conditions of 10 % equibiaxial stretch. Moreover, rat VSMCs subjected to 20 % cyclic stretch for 24 h displayed increased TGF-β1 mRNA expression and protein secretion. In this study, TGF-β1 stimulated expression of ECM components, outlining its role in vascular remodelling [76]. As TGF-β1 has been implicated in VSMC stretch-responsive proliferation, differentiation, and secretion of ECM components, it is plausible that this cytokine may be a biomarker of cyclic mechanical stretch in VSMCs.

#### Secreted EC factors

*In vivo*, paracrine signaling from nearby ECs may play a large role in VSMC response to mechanical stress. For this reason, VSMC-EC co-cultures or use of media from stretched ECs to culture VSMCs, may represent a more accurate system when trying to model cyclic strain *in vitro*. It has been previously shown that factors secreted by ECs are involved in VSMC stretch-responsive proliferation. An aortic EC-SMC co-culture model of high blood pressure increased VSMC proliferation, compared to static controls [77]. In contrast, researchers have found that media from pulmonary artery ECs subjected to 15 % elongation inhibited PASMC growth [78]. Specifically, the anti-angiogenic agent, thrombospondin-1, may be at least in part responsible for this inhibition of PASMC proliferation. Taken together, these results indicate that paracrine signaling from ECs may play a large role in VSMC responses to mechanical stress. In order to create a more accurate *in vitro* model of cyclic mechanical stress, it is important to take into consideration the factors released by ECs under these conditions.

## Conclusion

The extensive diversity of the data obtained from VSMCs exposed to mechanical stress indicates that there are many variables that can influence stretch-induced responses. The frequency, duration, degree and axis of stretch, along with the plate substrate, origin of VSMC (vein or artery) may affect downstream responses of stretched cells. In addition, factors such as cell passage number, content of culture media, and calibration of equipment, may also contribute the discrepancies reported for VSMC stretch response. Accordingly, while the available models may provide important information, it is crucial to identify and simultaneously measure key biomarkers that can confirm VSMC stretch-mediated responses. These biomarkers are paramount to elucidating how variations of stretch conditions influence stretch-induced VSMC responses.

## Abbreviations

AoSMC: Aortic smooth muscle cell
EC: Endothelial cell
ECM: Extracellular matrix
ERK: Extracellular signal-regulated kinase
FAK: Focal adhesion kinase
HASMC: Human aortic smooth muscle cell
IGF-1R: Insulin-like growth factor-1
MAPK: Mitogen-activated protein kinase
MHC: Myosin heavy chain
MMP: Matrix metalloproteinase
PASMC: Pulmonary artery smooth muscle cell
PKCδ: Protein kinase C δ
PVSMC: Porcine vascular smooth muscle cell
RASMC: Rat aortic smooth muscle cell
ROS: Reactive oxidative species
SHR: Spontaneously hypertensive rat
SIRT6: Sirtuin-6
SM: Smooth muscle
SMC: Smooth muscle cell
SRF: Serum response factor
TGF-β: Transforming growth factor-β
VSMC: Vascular smooth muscle cell
WKY: Wistar Kyoto
